# Powering the formation of alveoli

**DOI:** 10.7554/eLife.79651

**Published:** 2022-06-09

**Authors:** Qianjiang Hu, Melanie Königshoff

**Affiliations:** 1 Division of Pulmonary, Allergy and Critical Care Medicine, School of Medicine, University of Pittsburgh Pittsburgh United States

**Keywords:** lung, alveolus, mitochondria, activity, distribution, Mouse

## Abstract

Two cell types in the lung need specific numbers and distributions of mitochondria for alveoli to form correctly.

**Related research article** Zhang K, Yao E, Chen B, Chuang E, Wong J, Seed RI, Nishimura SL, Wolters PJ, Chuang PT. 2022. Acquisition of cellular properties during alveolar formation requires differential activity and distribution of mitochondria. *eLife* **11**:e68598. doi: 10.7554/eLife.68598.

In order to run their sophisticated machinery, cells need energy. This power is generated by the mitochondrion, an organelle that consumes oxygen to convert nutrients into chemical energy that the cell can use. Recent studies indicate that mitochondria are also involved in other tasks, such as signaling transduction and cell death (Chandel, 2014; Bock and Tait, 2020).

Accumulating evidence suggests that mitochondria allow lung cells to adjust to changes in external conditions (Aghapour et al., 2020). Notably, different cell types in the lung contain distinct numbers of mitochondria that are distributed differently depending on the cell’s role and energy demands (Cloonan et al., 2020). More recently, mitochondrial defects have been linked to aging and chronic lung disease, but how mitochondria contribute to lung development and repair remained unclear.

During development, the final part of the lung to form is the alveoli – the tiny sacs of air where the carbon dioxide produced by cells is exchanged for the oxygen they need to generate energy. The alveoli arise from a larger sac which is subdivided into smaller pockets separated by connective tissue known as the secondary septa (Figure 1A; Rippa et al., 2021; Schittny, 2017). For the alveoli to develop correctly, the multiple cell types that make up the secondary septa must coordinate how they move and proliferate. Now, in eLife, Pao-Tien Chuang and colleagues from the University of California, San Francisco – including Kuan Zhang as first author – report that the number and distribution of mitochondria in two cell types involved in forming the secondary septa is critical for alveolar formation (Zhang et al., 2022).

**Figure 1.**
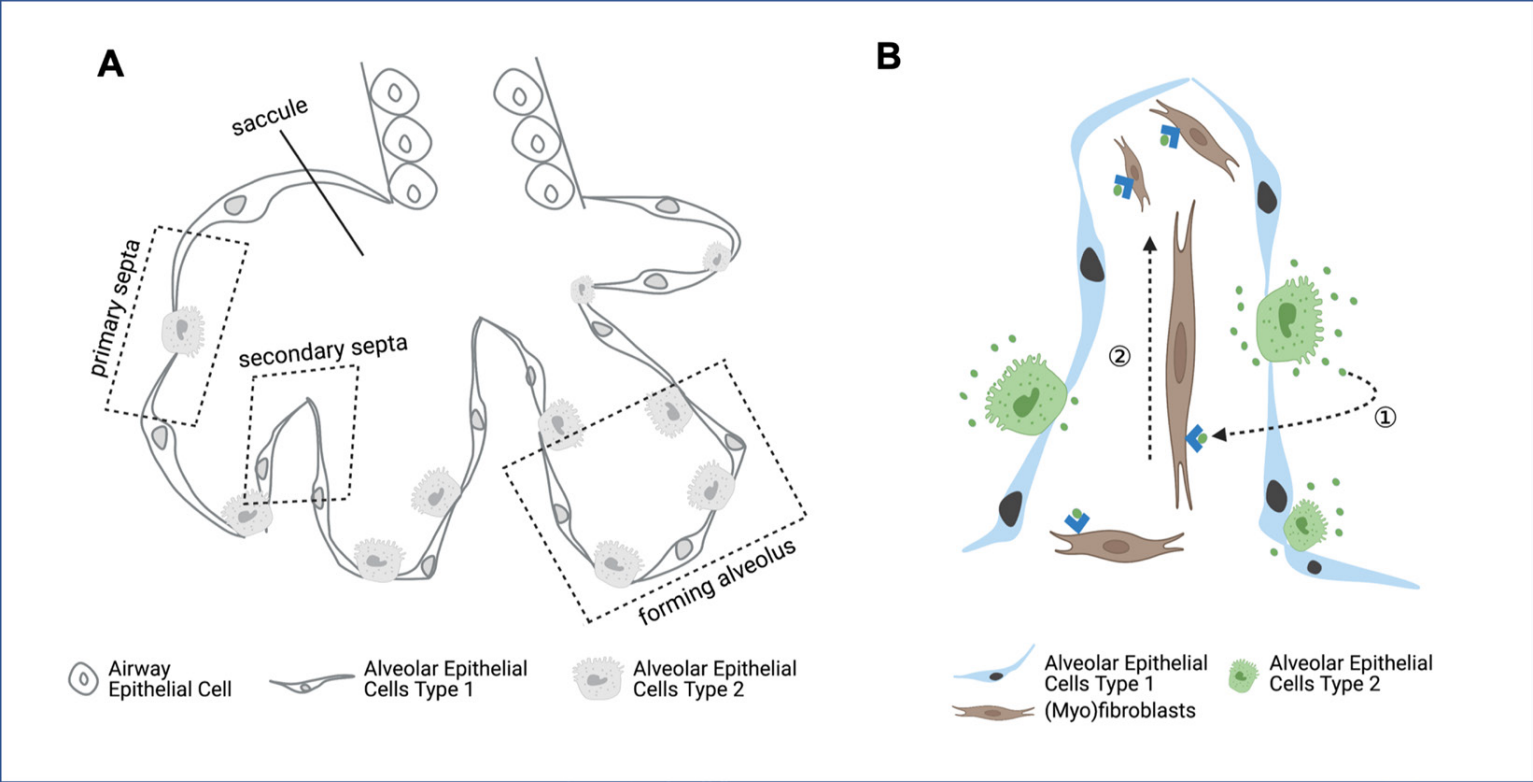
Formation of secondary septa in alveolar development. (**A**) During the final stages of lung development, cells forming the primary septa – the walls of the saccules – proliferate and migrate to form secondary septa, which divide the saccules into alveoli. The epithelial cells present at this stage of lung development are airway epithelial cells, alveolar epithelial cells type 1 and alveolar epithelial cells type 2. (**B**) Within the secondary septa are multiple cell types – including alveolar epithelial cells type 1 (blue), alveolar epithelial cells type 2 (green) and fibroblasts and myofibroblasts (brown) – that need to coordinate their movement and proliferation. Alveolar epithelial cells type 2 secrete platelet derived growth factor (PDGF, green dots), which binds to receptors (blue arrowheads) on the surface of fibroblasts and myofibroblasts (dashed arrow 1). This drives the proliferation of the myofibroblasts and fibroblasts (dashed arrow 2), which is required to form secondary septa.

Image credit: figure created using BioRender.

First, using immunostaining of mouse lung sections, Zhang et al. observed that mitochondria were unevenly distributed in alveolar epithelial cells and myofibroblasts, two types of cells important in the development of alveoli. In the epithelial cells, which secrete signaling molecules essential for the formation of secondary septa, mitochondria were found near the Golgi apparatus, an organelle that helps package proteins into vesicles prior to secretion. Meanwhile, in myofibroblasts, which migrate to help build the secondary septa during alveolar development, mitochondria were clustered around smooth muscle actin, a protein that allows cells to change shape and migrate. Hence, the distribution of the mitochondria in epithelial cells and myofibroblasts during lung development may respond to the role and energy demands of these cells.

Next, Zhang et al. used mice lacking a protein that regulates mitochondrial transcription to investigate how disrupting the activity and distribution of mitochondria in epithelial cells impacted the development of alveoli. They found that this disruption impaired the formation of secondary septa and reduced the number of myofibroblasts in the lung.

During secondary septa formation, the proliferation of myofibroblasts in the lung is driven by platelet derived growth factor (PDGF), a signaling molecule secreted by alveolar epithelial cells (Figure 1B; Boström et al., 1996). In these cells, PDGF is processed through the Golgi apparatus, where it is packaged into vesicles to be secreted. Zhang et al. had already determined that in these epithelial cells, the Golgi apparatus is surrounded by mitochondria. Now, they wanted to know whether disrupting mitochondrial activity in these cells would affect either the synthesis or the secretion of PDGF, thus reducing myofibroblast proliferation and resulting in a smaller myofibroblast population. The experiment revealed that alveolar epithelial cells containing defective mitochondria were able to synthetize PDGF, but could not secrete it, indicating that releasing PDGF is energetically costly.

In addition, Zhang et al. perturbed the activity and distribution of mitochondria in myofibroblasts to see how this affected lung development. They found that this stopped the myofibroblasts from migrating, further compromising alveolar formation. If a person’s alveoli fail to develop correctly, this could predispose them to chronic lung diseases such as chronic obstructive pulmonary disease (COPD) later in life. Importantly, the possible upstream causes of mitochondrial dysfunction in the developing lung remained unclear.

Zhang et al. hypothesized that the mammalian target of rapamycin (mTOR) signaling pathway might be involved, as it has been shown to regulate cell proliferation, nutrient balance and energy supplies during lung development (Land et al., 2014). Furthermore, there is also evidence to suggest that mTOR signaling coordinates energy consumption and mitochondrial activity in cells (Morita et al., 2015). To investigate the role of mTOR in the development of alveoli, Zhang et al. used a common genetic technique to remove a key protein in the mTOR signaling pathway from the lungs of mice. This resulted in lung cells having fewer mitochondria, which, in turn, impaired the formation of the secondary septa and the development of the alveoli.

Zhang et al. then repeated some of the experiments on human lung epithelial cells and fibroblasts grown in the laboratory. As expected, disrupting the number and distribution of mitochondria stopped the epithelial cells from secreting PDGF ligands, and compromised the migration of human lung fibroblasts.

Taken together, these findings suggest that mitochondrial defects can lead to errors in alveolar development and regeneration. In conditions such as COPD, alveoli are continuously destroyed and fail to regenerate. Indeed, Zhang et al. found that patients with COPD exhibited altered mitochondria and had lower levels of key mitochondrial proteins in their lung tissue than healthy individuals, further supporting a link between mitochondrial activity and lung regeneration.

This study demonstrates that alveolar epithelial cells and myofibroblasts require different mitochondrial distribution and activity for alveoli to form correctly. Further research could reveal whether these factors could also affect the roles of other cell types in lung development. A better understanding of how energy is used at different stages of alveolar formation could lead to new therapies for chronic lung diseases, such as COPD.
